# Some Design Considerations in Passive Indoor Positioning Systems

**DOI:** 10.3390/s23125684

**Published:** 2023-06-18

**Authors:** Jimmy Engström, Åse Jevinger, Carl Magnus Olsson, Jan A. Persson

**Affiliations:** 1 Sony Europe B.V., 223 62 Lund, Sweden; 2 Internet of Things and People Research Center, Department of Computer Science and Media Technology, Malmö University, 205 06 Malmö, Sweden; ase.jevinger@mau.se (Å.J.); carl.magnus.olsson@mau.se (C.M.O.); jan.a.persson@mau.se (J.A.P.)

**Keywords:** BLE, fingerprinting, indoor positioning, multilateration, RSSI, privacy

## Abstract

User location is becoming an increasingly common and important feature for a wide range of services. Smartphone owners increasingly use location-based services, as service providers add context-enhanced functionality such as car-driving routes, COVID-19 tracking, crowdedness indicators, and suggestions for nearby points of interest. However, positioning a user indoors is still problematic due to the fading of the radio signal caused by multipath and shadowing, where both have complex dependencies on the indoor environment. Location fingerprinting is a common positioning method where Radio Signal Strength (RSS) measurements are compared to a reference database of previously stored RSS values. Due to the size of the reference databases, these are often stored in the cloud. However, server-side positioning computations make preserving the user’s privacy problematic. Given the assumption that a user does not want to communicate his/her location, we pose the question of whether a passive system with client-side computations can substitute fingerprinting-based systems, which commonly use active communication with a server. We compared two passive indoor location systems based on multilateration and sensor fusion using an Unscented Kalman Filter (UKF) with fingerprinting and show how these may provide accurate indoor positioning without compromising the user’s privacy in a busy office environment.

## 1. Introduction

Indoor positioning of a user is somewhat of a holy grail within context-aware Internet of Things (IoT) systems. While GNSS [1] allows for accurate outdoor positioning, as long as we have a free Line of Sight (LoS) to GNSS satellites, locating a user indoors is still an open research area. The most-common indoor method today is fingerprinting [2], i.e., where a device compares recently collected signal strength values from various sources, such as WiFi access points and beacons, using Bluetooth Low Energy (BLE), with a reference database that is often kept in the cloud. Examples of vendors of such online databases are Ekahau and Skyhook. This is a robust method because it does not require much adaption or tuning as long as enough recorded data exist. Given the recent decrease in online storage and deployment cost, this method works well when supported by large cloud environments. However, it also comes with a significant loss of privacy for the end-user. For instance, in cases where the user wants to store a favourite place or a landmark or view his/her position on an indoor map, he/she needs to upload his/her current RSSI fingerprint to a server. This is in contrast to being outdoors, as a GNSS receiver, such as a mobile phone, passively listens to satellite communications and estimates the users’ location on the device. Consequently, the user’s privacy is preserved during outdoor GNSS use, while fingerprinting for indoor use typically does not allow this as it relies on large online databases of fingerprints.

There are many different methods that attempt to solve the indoor positioning problem, such as magnetic fingerprinting, which relies on a pre-recorded database of magnetic field measurements for an area [3,4], Pedestrian Dead Reckoning (PDR) [5], which uses accelerometer and gyro measurements to estimate the path of a user, the angle-of-arrival, where angular measurements based on the phase shift of signals from several beacons are used to estimate the user location [6], and Time of Flight (ToF) [4] measurements, which are often used within Ultra-Wideband (UWB) communication to estimate the distance between the user and known beacons with high accuracy. Similarly, WiFi-RTT [7] measures the round trip time to estimate the ToF. However, direct use of ToF measurements requires bidirectional communication between the device and beacons both for UWB and WiFi-RTT, although in the latter case, we are usually using access points supporting the Fine Time Measurement (FTM) extension to IEEE 802.11-2018 [8]. To avoid bidirectional communication, one may use, e.g., the Downlink Time Difference of Arrival (DTDoA) [9], where the device uses the time differences between received messages from nearby beacons to estimate its location in a similar way to how most consumer GPS calculates time differences between satellite signals. Still, such an approach also introduces new challenges, such as the need for a time-synchronised beacon infrastructure.

The Angle of Arrival (AoA) [6] is a method where the phase difference of a signal that hits an array antenna is used to estimate the angle between the beacon and locator. In an AoA configuration, the mobile device would transmit a signal picked up by several array antennas, thereby making it an active method. In another configuration, the Angle of Departure (AoD), an array antenna sends a signal that is picked up by the device. Only the AoD can be passive if we want the size of the mobile device to be kept reasonably small due to the required size of the antenna array, but that introduces other requirements, such as the time synchronisation of anchors, to avoid interference. Unfortunately, both the AoA and AoD have only recently been added to the Bluetooth SIG specification, Version 5.1, and are thereby not well supported in existing operating systems such as iOS or Android.

Multilateration [10] or trilateration are also methods that may be used for indoor positioning. They rely on the position of a device as calculated by using distance measurements between the device and multiple landmarks, such as BLE beacons. The distance estimates are either direct measurements of the distances or calculated from other measurements such as the signal strength, as in [11]. This method only requires known landmark positions to estimate the device location, making the data size several orders of magnitude smaller than fingerprinting. This also avoids the time-consuming data collection of signal strength values at the deployed location to build a reference database.

Methods that utilise signal strength measurements will be heavily affected by multipath and shadowing noise. Given that many methods utilise the 2.4 GHz frequency band, i.e., WiFi and Bluetooth, the amount of multipath and shadowing are very much dependent on the building, floor, and layout of the current room [12], since a large amount of the transmitted energy is reflected from surfaces such as walls, ceilings, and furniture.

Sensor fusion combines position estimates with sensor data from an Inertial Measurement Unit (IMU). The measurements from, e.g., MEMS gyroscopes are very accurate. An IMU for wearables, such as the Bosch BMI270, has an angular measurement error of ±0.4 % and a bias of ±0.5 dps. These IMUs generally estimate a device’s pose in the short term. However, they will accumulate bias over time, which needs to be mitigated. This bias can be reduced by using the signal broadcast from stationary beacons. A family of common sensor fusion methods is Kalman filters, such as the original Kalman filter [13], the extended Kalman filter, and the unscented Kalman filter by [14]. Other approaches use a particle filter [15] or a neural network combined with a Kalman filter [16]. Kalman filters are state-space models, defined by a transition model $x_{t+1}=Fx_{t}+Gw_{t}$, where the new state is estimated to be a linear transition operation from the previous state with an additional noise term $Gw_{t}$, and a measurement model $Y_{t+1}=Hx_{t}+v_{t}$, where the measurement is a linear transform of the current state $x_{k}$ with an additional noise term $v_{t}$. In indoor navigation scenarios, the state usually includes the current x, y, and heading in the local coordinate system for the building, but may also include the first-order derivative of the state, i.e., movement and turning speed. The next state is then predicted given sensor data such as the IMU. Finally, the state is updated by comparing the predicted state with other sensor measurements, such as distances to beacons nearby, and then correcting the current state information. Particle filters also estimate the state of a system. However, instead of applying a linear operation on a single state, the state is represented as a distribution based on many particles. All particles have their own state estimate, and the next state is predicted based on the current state. The observation at time *t* is used to weigh the particles’ likelihood, which is then resampled according to their probability. In general terms, the particle filter allows the estimation of more complex distributions, such as multimodal distributions, compared to Kalman filters, but at the cost of computational resources.

For some of the methods mentioned above, it is either needed or required to use bidirectional communication with either other local devices or the server, thereby requiring the device to transmit information. Suppose a person’s location data are available for a third party, e.g., by letting a server compute the user location based on the RSSI values. In that case, it can also be used to infer sensitive information such as a user’s health, religion, gender, and sexual orientation [17], which may result in privacy violations, for instance physical threats. Location data can also be used for the de-anonymisation of an individual, as multiple locations can be combined into unique fingerprints. It has been shown by DeMontjoye et al. [18] that location data are highly discriminative and as few as four coarse location data points can identify 95% of the users. Considering the growing number of personal data breaches over the last decade [19], where some breaches expose billions of personal data records, there is an imminent need for methods that reduce the amount of online data that can be used for de-anonymisation of users.

We identified a lack of literature focusing on comparative studies for passive positioning methods based on the same dataset and environment. This is also evident in the article by Potortì et al. [20], where the EvAAL framework was introduced for evaluating the accuracy of indoor positioning methods. This is the framework used by the yearly IPIN competitions. However, there is a lack of BLE-based datasets for evaluation. Considering that the majority of the papers published evaluate fingerprinting, multilateration, sensor fusion, or a combination thereof, we wanted to see how fingerprinting and multilateration compare with sensor fusion using the exact same dataset. The choice of the dataset is important, as indoor positioning is quite complex and highly dependent on the experimental setup, and results from one study are hard to compare directly to another. By adequately evaluating the methods, especially by using data collected in an office during busy hours, we contribute to the understanding of the potential to increase privacy by shifting to completely passive methods under real-life conditions, which inherently have lower privacy-invasive characteristics.

This paper investigated the tradeoffs between using a passive on-device approach versus an active online-based approach for indoor positioning. Throughout the paper, we refer to communication as *passive* if it only listens to broadcasts and *active*, where bidirectional communication is needed. We implemented fingerprinting, multilateration, and finally, a sensor fusion model based on an Unscented Kalman Filter (UKF) and compared their strengths and weaknesses on the same dataset.

### Previous Work

Over the last decade, fingerprinting has become the dominant method for estimating indoor positions. A large part of the research on indoor positioning is related to fingerprinting methods. In order to quantify the amount of research performed using each method, we used EBSCO [21] to search for papers that included the terms *indoor* and either *position* or *location* in their title, abstract, or keywords. Then, we categorised the 3728 entries by searching the metadata to identify if the paper should be associated with *fingerprinting, multilateration, ultra-wideband, machine learning, sensor fusion* or *other*. The logic for the associations follows Table 1, where the category is defined by the first match in the table.

Care was taken to allow many different variants of spelling and naming, such as “fingerprint”, “knn”, and “cluster” to be assigned to “fingerprinting”. We chose this categorical classification because we wanted to see how many papers focused on both fingerprinting and multilateration, as these are methods that can be applied to the exact same measurements if we are using BLE beacons. If a paper did not address both, we moved out all ultra-wideband-related articles to a separate category, as UWB usually is based on either ToF or TDoA measurements or, sometimes, both. We prioritised labelling multilateration and fingerprinting for the remaining articles before their machine-learning-based approaches. The reason for this is that many machine learning approaches utilise fingerprints, so these categories overlap, but if the paper utilised both fingerprints and machine learning, we found it more suitable to assign the latter category. Finally, the sensor fusion approach captured papers discussing particle filters, Kalman filters, sensor fusion, and unscented Kalman filters.

**Figure 1:**
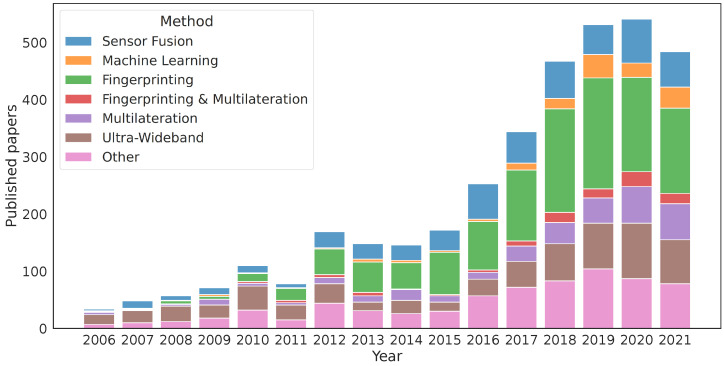
Papers published related to indoor positioning.

For an overview of these technologies, Deak et al. [22] gave a survey on many different indoor positioning approaches, including device-free methods that do not require the user to carry an electronic device, while [23] focused on learning-based fingerprinting location methods. Note that *active* and *passive* in [22] refer to whether the user carries an electronic device or not. Deak et al. [24] gave a survey on smartphone-based methods, noting that there is a lack of standardised procedures for positioning accuracy evaluation and that real-life performance is usually significantly worse than the 1–2 m accuracy reported in some studies. One of the reasons mentioned is that the experiments are often performed in a small area filled with beacons. Duan et al. [25] relaxed this requirement and defined *passive* as “application free” in their approach, where they located a user by measuring the data rate between the user device and access points in a WiFi network. These definitions differ from ours, where we define *passive* as whether the user device transmits data. Some papers covered hybrid approaches between fingerprinting and multilateration, and these were classified into separate categories. Figure 1 shows the distribution of articles per year, and Table 2 shows the total. It is evident that fingerprinting is overwhelmingly dominant, corresponding to 32% of the total amount of publications addressing indoor positioning. However, fingerprinting databases are quite large, making it more convenient to store the databases and run positioning calculations online, requiring clients to upload data such as RSSI measurements.

One approach to overcome or reduce the privacy concern is to rely on dummy location requests generated randomly within a certain distance from the user [26]. This hides the actual user location for one location request. However, this approach is susceptible to side-channel attacks when there are several requests, and the attacker has some side-channel knowledge of the likelihood of the specific trajectory at each location [27].

Another approach is to apply cryptography to preserve the privacy of both the user and the server. There have been attempts to use different encryption mechanisms to enhance fingerprinting privacy, with [28] being one of the most-referenced. The cipher is homomorphic in the Paillier crypto scheme, which enables computations such as addition and subtractions on encrypted data without decrypting them first. In a positioning scenario, the user device sends encrypted landmark fingerprint samples from his/her environment to the server, which computes the distances between known landmarks in its database and the encrypted fingerprints. The user device can then decrypt the result from the server to determine the position. Paillier’s crypto scheme is used in the Privacy-preserving WiFi Fingerprint Localisation scheme (PriWFL) by [29] and Lightweight Privacy-Preserving Scheme (LWP) [30]. Li et al. focused on four types of attack patterns, as seen in Table 3. However, Reference [31] published an attack on the PriWFL scheme, where specially crafted messages sent to the server could reveal the whole landmark database. Overall, it is hard to design a generic encryption scheme that thwarts all types of known and unknown attacks. Consequently, published methods are usually narrow in scope, focusing on specific use cases [32].

The scope of our study is limited to methods that use passive communication, thereby eliminating the location privacy attacks given in Table 3, as no information is ever transmitted from the user device. The secrecy of the server database was not considered in this research as the database consists of only beacon IDs and their corresponding local x and y locations in meters, and the beacons have no relation to other infrastructure in the buildings.

## 2. Materials and Methods

To investigate the tradeoffs between using different passive, on-device approaches for indoor positioning, we used two of the most-common methods, fingerprinting and multilateration, as a baseline for BLE-RSSI-based positioning. These were then compared with a passive sensor fusion method based on the BLE signal strength and inertial data from a mobile device. One important aspect of our experiment was that the data collection was performed in a busy office environment, representing actual, real-life conditions. The 2.4 GHz spectrum, where Bluetooth and WiFi are situated, is quite crowded, especially during busy workdays in an office. In order to compare the methods, the measured beacon data and device sensor data were collected once, and the three different methods were subsequently applied offline. From a conceptual point of view, the processes of collecting and using BLE or WiFi for fingerprinting are very similar. Both use the 2.4 GHz band and can be captured with consumer devices such as mobile phones using the same antenna, and we collected the signal strength at known locations. However, through the iBeacon [33] protocol, BLE has additional information attached, such as the beacon ID and transmission strength, which allows the client to estimate the distance based on the Received Signal Strength Indicator (RSSI). In Table 4, we show an overview of the three methods configured only to use passive communication.

An important difference between fingerprinting and multilateration is their sensitivity regarding radio signal shadowing, where large objects such as walls or furniture can considerably reduce the received signal strength. This may be a benefit when using fingerprinting, as it increases the uniqueness of a specific location, making the signal strength significantly different from other locations. For multilateration, shadowing is problematic, as the most-used distance estimation formula, the log distance path loss model (see Section 2.2), only has a single coefficient to adjust the path loss to a specific environment. Sensor fusion can utilise, e.g., an IMU to stabilise the path, reducing the effect of severe local shadowing.

Multipath fading, where the same broadcast signal interferes with itself, can increase or decrease the received signal strength. However, if we are utilising a carrier frequency of 2.4 GHz, the half wavelength is about λ/2=6.3 cm. This means that our received signal strength can change considerably by moving the receiver 6 cm. Puccinelli and Haenggi et al. [34] analysed multipath fading in sensor networks, but with a carrier frequency of 433 MHz. Due to the nature of multipath fading, it is often modelled as noise instead of creating very detailed local propagation models. Thus, in contrast to shadowing, multipath fading is a problem for all three methods, although, as before, the stabilising properties of sensor fusion reduce the effect.

Given the common use of WiFi RSSI fingerprinting, we note that BLE RSSI fingerprinting is very similar to WiFi fingerprinting. The main difference between WiFi access points and BLE beacons is that the BLE beacons are usually more densely populated than the WiFi access points. Lindemann et al. [32] published a study comparing the differences in WiFi and BLE fingerprinting accuracy, concluding that the higher density of the BLE beacons resulted in higher location accuracy. Sadowski and Spachos [35] concluded in their experiment that they gained very little by using more than five beacons to estimate a location using Non-Linear Least Squares (NLLS).

For this study, we assumed that beacon locations either exist in an online database or that there is a fingerprinting database of RSSI values that can be distributed to the client before running the indoor location algorithms on a device. It should be noted that this method does not provide complete user anonymity. The user must download the beacon location database, so his/her IP address will be revealed. This could be mitigated in several ways, e.g., by preloading the data in the device, downloading the database through a trusted proxy or Virtual Private Network (VPN), using encrypted peer-to-peer services such as Tor, or by downloading many simultaneous location databases and, thereby, hiding the actual location, among others. As this study was limited to establishing the location performance once the landmark database is known, we did not take such measures in our experiments. A summary of the data use in the methods can be found in Table 4.

### 2.1. Fingerprinting

Fingerprinting requires collecting RSSI data from many known locations within the building, which is labour-intensive. A dense collection of fingerprints improves the location accuracy, as our k-Nearest Neighbour (k-NN) algorithm [36] returns the sample with the smallest Euclidean distance compared to the input fingerprint. Due to the magnitude of data needed to achieve good location accuracy [37], the database is often stored online and then queried to find the closest matches to the user-provided RSSI fingerprint. The data storage requirements grow linearly with the amount of collected fingerprinting data, but it may be reduced by calculating the mean or median value of the RSSI samples on a grid applied to the ground truth locations. Then, the granularity of the grid and the number of beacons decides the fingerprinting database size. In our case, we set the grid size to 1 × 1 m. We define the RSSI of a landmark as $l_{t,i}$ for time *t* and landmark ID *i*. The input values, where *X* is a *T* by *I* matrix,

(1) $$X=\left[\begin{array}{cccc} l_{0,0} & l_{0,1} & \cdots & l_{0,I-1}\\ l_{1,0} & l_{1,1} & \cdots & l_{1,I-1}\\ \vdots & \vdots & \cdots & \cdots \\ l_{T-1,0} & l_{T-1,1} & \cdots & l_{T-1,I-1} \end{array}\right]$$

and the target labels *Y* are a *T* by two matrix, containing the *x* and *y* locations of the fingerprinting grid:

(2) $$Y=\left[\begin{array}{cc} x_{0} & y_{0}\\ x_{1} & y_{1}\\ \vdots & \vdots \\ x_{T-1} & y_{T-1} \end{array}\right]$$

where *T* is the number of time steps and *I* is the number of landmarks.

When calculating k-NN using the landmark matrix (1), we need to remove missing values, as the KNeighboursRegressor cannot handle these. This can be performed in several ways, such as deleting landmarks with missing values or imputations. In location scenarios, most landmarks have missing values at some point, making the deletion of these landmarks an inefficient strategy. The many missing values are caused partly by the low duty cycle of the beacons of 1 Hz, making it likely to miss a broadcast due to interference, but it can also be caused by beacons being too far away from the receiver. A higher sampling frequency than 1 Hz in the receiver will result in more time windows with missing data. The RSSI values were sampled in a grid using a median filter over a sliding time window to impute missing values. If there were still missing values, these were imputed with a constant RSSI; however, the size of the sliding time window and the imputed constant RSSI need to be tuned. Localisation using the fingerprinting database was performed by first locating the k closest matches in the database, based on the Euclidean distance with uniform weights, and then calculating the mean of the associated target labels, in our case the x and y position of the cells in the grid. The method was implemented as KNeighboursRegressor in Scikit-Learn [38].

### 2.2. Multilateration

Multilateration uses distance measurements between the device and known landmark positions, such as beacons, to estimate the user’s location. In contrast to the extensive data collection needed for fingerprinting, this approach only requires known landmark positions. This is often performed using Gauss–Newton iteration to solve the non-linear least squares equations that define the multilateration problem. However, as we lacked direct distance measurements, we converted the RSSI measurements to true range estimates as in [39] by applying the log-distance-path-loss model [40].

(3) $$PL={PL}_{0}+10\gamma \log_{10}\frac{d}{d_{0}}+X_{g}$$

where PL is the measured total path loss in dB, $PL_{0}$ is the measured path loss at the reference distance $d_{0}$, γ is a path loss exponent, and $X_{g}$ is a Gaussian random variable with zero mean, representing fading of the signal. The variable $X_{g}$ has a significant effect on the estimated distance, and the standard deviation of the term is roughly 2–3 dB in 2.4 GHz channels [41,42], but heavily dependent on the environment. This can translate to tens of meters in the calculated distance difference between consecutive readings between stationary transmitters and receivers.

If we assume that $PL_{0}$ is measured at 1 m and that our shadowing noise $X_{g}$ is Gaussian with zero mean, then $d_{0}=1$. Solving for *d* gives us

(4) $$d=10^{\frac{PL-PL_{0}}{10\gamma }}$$

or, if we prefer to use RSSI values instead of path loss:

(5) $$d=10^{\frac{RX_{d}-RX_{0}}{10\gamma }}$$

where $RX_{0}$ and $RX_{d}$ are the measured signal strengths in dBm at one meter and *d* meters.

Once we had the estimated ranges, we estimated the position by optimising a cost function using the Limited-memory Broyden–Fletcher–Goldfarb–Shannon Bound algorithm (L-BFGS-B) [43], where the constraints are the physical dimensions of the room. We define the vector $U_{t}=\left[x_{t},y_{t}\right]$ as an estimate of the current user position at time *t* and the N × 2 matrix *B* as the known beacon positions:

$$B=\left[\begin{array}{cc} x_{0} & y_{0}\\ x_{1} & y_{1}\\ \vdots & \vdots \\ x_{N-1} & y_{N-1} \end{array}\right].$$

Finally, we define a measurement vector *D*, which is the set of collected RSSI values converted to distances, $D=10^{\frac{PL_{0}-R}{10\gamma }}$, where $R=[rssi_{0},rssi_{1},\ldots ,rssi_{N-1}].$ We define our cost function cf(U,B,D) for the non-linear optimisation target as

(6) $$\begin{array}{cc} \underset{U}{arg min}cf\left(U,B,D\right) & =\sum_{i=0}^{N-1}\frac{\left(||U_{i}-B_{i}|{|}_{2}-D_{i}\right)}{D_{i}W} \end{array}$$

(7) $$\begin{array}{cc} \mathrm{where}\;W & =\sum_{i=0}^{N-1}1/D_{i} \end{array}$$

The cost function represents the difference between the estimated distance to beacons using RSSI values and the estimated distance to beacons using the user location vector $U_{t}$. The inverse of the distance measurement is used to weigh the values, as we have lower confidence in measurements from beacons far away, as the amount of noise in the RSSI measurements increases with distance.

### 2.3. Sensor Fusion

When there are multiple noisy sources of information, such as sensor readings, we may apply sensor fusion to achieve a result that has less noise than each source separately. The Kalman Filter (KF) and its variants, the Extended Kalman Filter (EKF) and Unscented Kalman Filter (UKF), are common methods for sensor fusion. Whereas the Kalman filter is an optimal linear estimator, the EKF and UKF allow non-linear functions representing the predicted next state. The UKF allows the capture of higher-order moments than the EKF and removes the need to calculate the Jacobian of the transfer function, as it relies on sampling using the unscented transform instead. The approach used in this paper was the same as the specific configuration in [44], where the beacon locations are known. The unscented Kalman filter fuses sensor data by combining a Pedestrian Dead Reckoning (PDR) algorithm based on inertial sensor data with RSSI measurements. The PDR was used by Team Sony in IPIN 2018 [45]. In essence, Kalman-based sensor fusion consists of cyclic prediction and update stages, where in this case, the PDR was used to estimate the trajectory of the user to predict a new state, and then, the beacon distance measurements were used to update the state.

This type of sensor fusion is more resilient to noisy data, as it continuously tracks the user position. In contrast, multilateration and fingerprinting are memoryless in their basic forms, making them more sensitive to outliers. This may often occur in indoor location scenarios due to the very noisy nature of the received signal strength, especially if only a small number of received RSSI values exist.

#### Filter Configuration

The initial state is defined as $x_{0}=\left[x,y,\theta \right]=\left[0,0,0\right]$, where the first two elements represent our position and θ is the direction. As we have no information regarding the starting position or direction, the matrix *P*, which represents the covariance of the state, was set to

$$P=\left[\begin{array}{ccc} 100^{2} & 0 & 0\\ 0 & 100^{2} & 0\\ 0 & 0 & \pi^{2} \end{array}\right]$$

Our process noise was set to

$$Q=\left[\begin{array}{ccc} 0.02 & 0 & 0\\ 0 & 0.02 & 0\\ 0 & 0 & \frac{\pi }{4} \end{array}\right]$$

An update with a single beacon measurement uses a measurement covariance matrix:

$$R=\left[\begin{array}{cc} d^{2}\left(e^{\frac{\sigma^{2}}{2\eta^{2}}}-1\right)e^{\frac{\sigma^{2}}{2\eta^{2}}} & 0\\ 0 & {\left(\frac{\pi }{6}\right)}^{2} \end{array}\right],$$

where *d* is the estimated distance based on the log distance path loss Equation (4). The signal strength measured at a certain location is affected by many factors, such as user location, distance to the beacon, and how people are walking around, among many other things. We used a single path propagation model and, thus, a single σ and η for the whole experiment, knowing that this is a simplification of a complex environment. A more complex filter could be set up to not just track the user state, but also the location and covariance of all beacons at the expense of more calculations and memory usage. This type of configuration was used in [44] to locate beacons automatically.

If we assume the same σ and η, which represent environment noise and path loss for all beacons, most of the expression can be replaced with a constant *K*, and we can simplify the expression to

$$R=\left[\begin{array}{cc} d^{2}K & 0\\ 0 & {\left(\frac{\pi }{6}\right)}^{2} \end{array}\right]$$

When there are N simultaneous beacon distance measurements $d_{1}\ldots d_{N}$, these can be used in the update stage of the filter by configuring the measurement noise covariance as

$$R=\left[\begin{array}{cccc} d_{1}^{2}K & \cdots & 0 & 0\\ \cdots & \cdots & 0 & 0\\ 0 & 0 & d_{N}^{2}K & 0\\ 0 & 0 & 0 & {\left(\frac{\pi }{6}\right)}^{2} \end{array}\right]$$

In the UKF, certain parameters are needed for configuring the sigma points used in the unscented transform. These parameters are kept to the same values as recommended in [46], with κ=0, β=2, and $\alpha =10^{-3}$.

### 2.4. Experimental Setup

Within an office environment, 12 × 72 m in size, we mounted 20 BLE beacons at 1.2 m above the floor. These beacons had a broadcast frequency of 1 Hz, and the received signal strength at 1 m was –65 dBm, i.e., $PL_{0}=-65$. Specifying the measured signal strength on the receiver side at 1 m removes the need for modelling specific antenna coefficients on both the transmitter and receiver, and this parameter is commonly named the *TX power* in the iBeacon standard [33]. A constraint of 1 Hz in beacon advertising frequency is required in scenarios where the infrastructure is supposed to be battery-powered for years, using only a small coin battery, such as a CR2032. Increasing the advertising frequency to 10 Hz would increase the accuracy [47], but at the expense of replacing all the batteries in the beacons every few months. We sampled the BLE RSSI and IMU data using a Sony Xperia 8 Android phone running Android 9 and using a Qualcomm Snapdragon 630 chipset. The data collection was performed by walking around in the environment for 22 min at noon on 4 December 2020. This was a time when the office was fairly busy. The IMU data consisted of accelerometer, gyroscope, and magnetic field measurements, i.e., 9 Degrees of Freedom (DoF) were sampled at 50 Hz. Once the data collection was completed, the results were offloaded and processed on a computer. The collected data were split into a training and a testing dataset, where the first 18 min were used for training and the last 4 min for testing. As there was very little difference in time between the collected training and testing datasets, we avoided the effect of slow-moving shifts in signal strength caused by, e.g., the different amount of people at the office or rearranged furniture.

The window size for grouping measurements into feature vectors was set to 2 s, and the imputed distance measurements required by fingerprinting were set to 10.5 m. This distance equals an RSSI value of −106 dBm, which is below the receiver’s noise threshold of approximately −105 dBm. The purpose was to impute values that were not captured, with a distance just outside the noise threshold.

Multilateration requires at least 3 valid RSSI measurements from different beacons to calculate a position. This is in contrast to fingerprinting, where we can estimate a location from a single RSSI measurement that is matched against the fingerprinting database. To enable the calculation of more locations using multilateration, we applied data aggregation in 2 s time slices when running multilateration. No aggregation was performed for sensor fusion or fingerprinting.

Regarding human mobility in the area, about 25 people were working at their desks within the experimental area, all utilising WiFi connections with their laptops and some also using Bluetooth headphones, thereby occupying part of the bandwidth of the 2.4 GHz spectrum shared with the BLE beacons.

Given these experiment conditions, the device cannot capture all advertised beacon messages. This can be the result of several reasons, such as:

- The signal may be below the noise threshold of the receiver.
- Other units in the 2.4 GHz spectrum, such as other beacons, Bluetooth, or WiFi devices, may interfere.
- The Bluetooth stack of the capturing device may be unable to process all broadcast messages.

Boosting the TX power of beacons or the sensitivity of the receiving antenna would possibly reduce the amount of missed BLE messages, but we did not conduct such experiments as we used off-the-shelf hardware components. In our experience and based on the manufacturer’s claim of range, this type of beacon typically has a range between 10 and 30 m, depending on the environment and receiver used.

As an approximation, we can use the path loss Equation (4), the provided TX of −65 dBm, and an assumed environment constant γ=2.8, i.e., we assumed the same path loss constant as in [42], which, as we also did, placed both receiver and transmitters at 1.2 m height. We applied the path loss formula, but added the constraint −105 dBm as the noise floor of the receiver:

(8) $$\begin{array}{cc} 105> & 65+10\times 2.8\log_{10}d \end{array}$$

(9) $$\begin{array}{cc} d⪅ & 27 \end{array}$$

Under these assumptions, beacons will not be heard at distances d≈27 m or more, due to the path loss. Considering that the total height of the map was roughly 70 m, we should expect to only receive a fraction of the transmitted messages and almost never more than 10 beacons at the same time. For the signal to reach 70 m in these conditions, our signal strength would have to be boosted by *B* dB:

(10) $$\begin{array}{cc} 105> & \left(65-B\right)+10\times 2.8\log_{10}70 \end{array}$$

(11) $$\begin{array}{cc} B⪆ & 12 \end{array}$$

As WiFi shares the same 2.4 GHz antenna and receiver module as BLE, it was turned off during the study. Otherwise, the communication methods were time slotted, reducing the amount of time BLE had for scanning nearby beacons.

To study the effect of the missed captured messages, augmented RSSI measurements were added to the dataset by drawing samples from the log-normal distribution $X_{g}$ in Equation (4).

**Figure 2:**
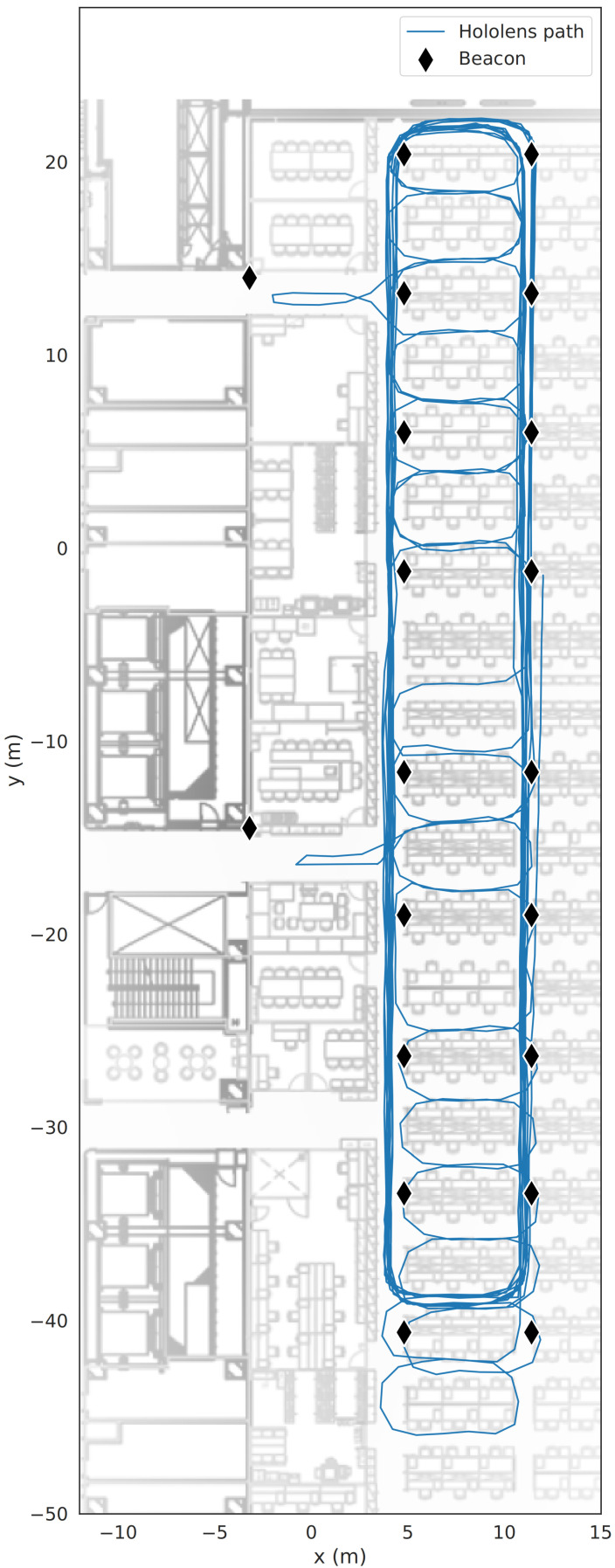
Ground truth using HoloLens.

The ground truth data of the *x* and *y* location of the user were collected simultaneously by using the Microsoft HoloLens, as seen in Figure 2. This method of collecting the ground truth gives a location error of less than 0.1 m. Other studies, e.g., by [48], estimated a relative positioning error of 0.016 m using the HoloLens. These were used as the reference data for fingerprinting and to estimate the positioning error when evaluating the methods.

The variance of the Gaussian shadowing noise $X_{g}$ was measured by placing a phone on top of a table in the middle of a room, far away from the walls, to avoid multipath propagation as much as possible. We had 707 BLE beacons available and used all these to calculate the variance of the RSSIs. Even though all beacons were not in the exact same spot, the shadowing noise parameter $X_{g}$ that we wanted to estimate was not distance-dependent; thus, we used the pooled variance:

(12) $$s^{2}=\frac{\sum_{i=1}^{k}\left(n_{i}-1\right)s_{i}^{2}}{\sum_{i=1}^{k}\left(n_{i}-1\right)}$$

where $s_{i}^{2}$ is the RSSI variance for the *i*th beacon and $n_{i}$ is the number of samples for the *i*th beacon. Using Equation (12), the estimated shadowing noise was $X_{g}=N\left(0,20.5\right)$.

While collecting data in the experiment, the user was always moving without any movement or turning speed restrictions. The movement and turning speed were continuously estimated by the PDR and UKF. This problem was more challenging than a stationary user, but provided a more realistic scenario. Even though a stationary user may move his/her hands and arms, it would require very specific motions to make the PDR detect this as a walking pattern, so a stationary user moving his/her arms is not as much of a problem as a walking user that simultaneously is moving his/her arms and hands unrelated to the actual walking motion. An added difficulty comes from the beacons only broadcasting once per second, which means the user will have moved quite far between the RSSI readings. The user had slightly different restrictions while collecting the training and testing data. When collecting the training data, the user was not allowed to enter the two horizontal corridors, while he/she was allowed to do so when collecting the testing data. This was to simulate a scenario where most, but not all, of the area was covered by the training data, which we deemed more realistic than having 100% coverage, especially if we consider larger installations.

The data were split into a training and testing dataset, where the first 4/5 of the data collected were used as the training data and the last 1/5 as the testing data. We ran the k-NN using k=14. Processing was performed in Python 3 [49] and supporting packages such as Pandas [50], Numpy [51], Scikit-Learn [38], and Matplotlib [52]. The UKF and PDR are proprietary implementations in Rust [53] and C [54].

## 3. Results

Fingerprinting was, as mentioned in Section 2.4, implemented by a k-NN classifier, which was trained on 80 % of the dataset and then split by timestamps. There was no information added to the k-NN classifier regarding the beacon locations, and it was purely based on the sampled RSSI values and the ground truth of the user position, defined by the HoloLens data capture. Given that the beacons broadcast their iBeacon messages with only a 1 Hz frequency, there were only a few samples to use for querying the database unless we aggregated the RSSI values over several seconds. This made the fingerprinting method sensitive to outliers in the test data, even if the training data were thoroughly collected.

In this experiment, there were 2933 raw RSSI samples available as training data. We note that this represents only a fraction of the broadcast messages, which we estimated to be ≈26,400 during the time frame. In other experiments, we saw that the mobile phone used in this particular experiment was unable to process more than 5–10 BLE messages per second, compared to other models that seem to be able to capture above 100 messages per second. We attributed the differences to the processing capabilities and Bluetooth stack running in mobile phones. Given the saturation of the number of processed messages, adding more beacons to the environment would likely not improve the accuracy of the system, which is in line with previous studies [35,55], where the authors in independent experiments saw little increased positional accuracy if the number of BLE beacons that could be sampled at a given time exceeded 6–8 beacons. To evaluate the possible accuracy improvement given a more capable Bluetooth stack, the augmented dataset added measurements with the same noise characteristics as the already collected data; see Section 3.1.

After aggregation into two-second time windows for multilateration, there were still 1537 samples recorded in the database. This reduction was mainly caused by replacing multiple RSSI values from a single beacon with the mean RSSI value. Multilateration only requires a list of beacon IDs, their position, and an estimate of γ. No such aggregation was necessary for sensor fusion or fingerprinting.

**Figure 3:**
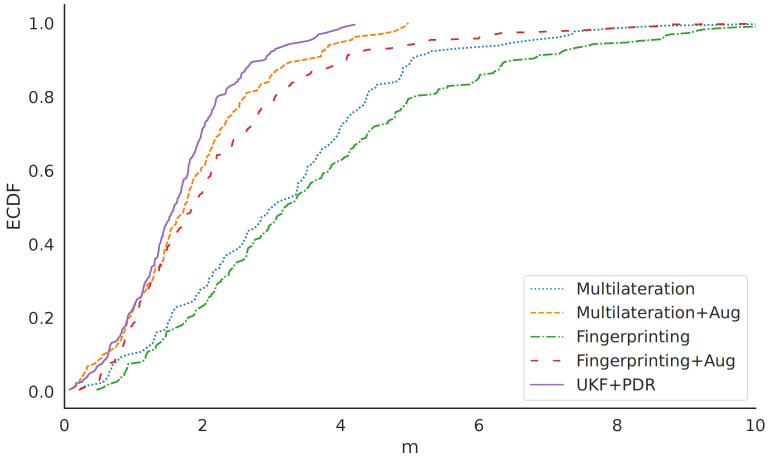
CDF of the positioning errors in meters.

The accuracy of multilateration is slightly higher than fingerprinting using k-NN, as we see in the Empirical Cumulative Distribution Functions (ECDFs) in Figure 3. At 80% confidence, the errors of fingerprinting were less than 5.1 m, while the multilateration errors were less than 4.4 m at the same confidence level. Although fingerprinting had similar accuracy to multilateration, the method requires substantially more effort to collect and store data. Of particular relevance is that the sensor fusion approach using UKF+PDR outperformed both methods by a large margin, with an 80 % confidence of 2.2 m.

### 3.1. Data Augmentation

We suspect that the inability of the mobile device to process all BLE transmissions had a great effect on the positioning accuracy. To study the effect of data starvation, we augmented the dataset by adding simulated RSSI measurements with the same amount of noise as the already collected data. The effect of the data starvation is apparent in Table 5, when we compare the positioning methods with and without augmented data. The augmented dataset represents a perfect environment where all BLE messages are received as long as the beacons are close enough to the receiver. In the collected dataset, all beacons were missing data at some point. In the augmented dataset, for each missing RSSI value, another RSSI was simulated instead unless the simulated RSSI was lower than −105 dBm, which was below the receiver’s sensitivity in the device and, consequently, treated as missing data. The augmented data improved the multilateration and fingerprinting datasets with an 80% CDF of 4.4 → 2.7 m and 5.1 → 3.0 m, respectively.

However, even when augmenting the data, neither multilateration nor fingerprinting surpassed the accuracy of the sensor fusion that only used the sampled data; see Table 5.

## 4. Discussion

In this paper, we set out to address some design considerations in an indoor position system. The presently dominating and robust approach of fingerprinting requires little adaption or tuning if there is enough recorded data, but has implications in terms of end-user privacy. Given the sensitive nature of location data, comparing fingerprinting accuracy with the alternative methods of multilateration and sensor fusion that handle data differently to infer the user location becomes relevant. Although all can technically run on an embedded device, the fingerprinting method in practice often uses online databases and computation. This is because fingerprinting databases tend to become larger than what is suitable for embedded devices, as the databases may span tens or hundreds of MBs. An example is the UjiIndoorLoc [56] dataset of 42 MB, which was used in the indoor positioning competition at IPIN 2015 [57]. In comparison, a beacon database used for multilateration for the same location would require less than 10 kB. The accuracy that fingerprinting can deliver thus comes at implied costs that may be too great for many embedded devices in context-aware IoT systems. The result is that fingerprinting systems often use active communication with a server that computes the location from fingerprinting data, such as Skyhook [58], Google Geolocation API [59], and Combain [60].

**Figure 4:**
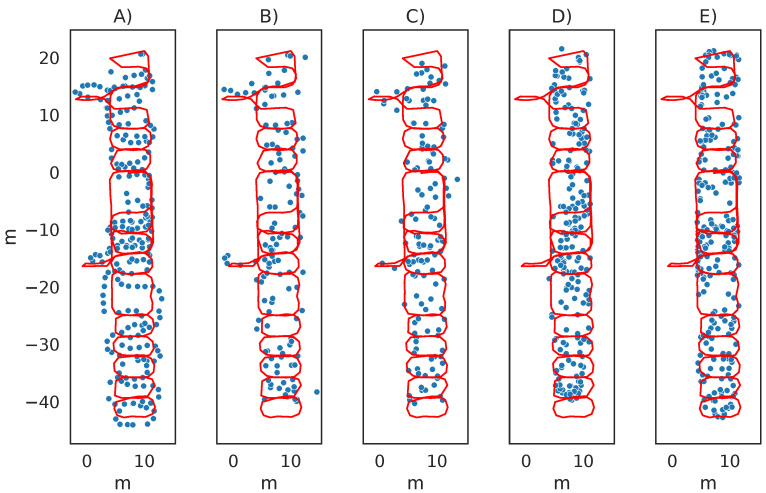
Estimated user positions. (**A**) UKF + PDR, (**B**) multilateration, (**C**) multilateration + augmentation, (**D**) fingerprinting, and (**E**) fingerprinting + augmentation. Ground truth is the red path and the blue circles are the estimated positions.

We still recognise that one argument for using fingerprinting instead of multilateration is that the positioning accuracy is often higher in such systems, as exemplified by [61]. One explanation for this is that the measured fingerprints include the distortions of the beacon signals at that specific location. In contrast, ranging-based methods, such as multilateration, are parametric with only a rough approximation of the signal propagation conditions. However, fingerprinting requires the sampled fingerprints to cover all possible locations. In our experiment, some parts of the test data were collected outside the path included in the training data. As a result, that part of the test path was completely void of any estimates needed for accurate fingerprinting results, as seen in Figure 4, where a small part of the ground truth was collected in two of the corridors at the far left of the map. In the conditions of our experiment, with infrequent beacon advertisements similar to how environments in practice are likely to be set up to preserve beacon lifespan, multilateration had similar accuracy as fingerprinting while only requiring the reference positions of the beacons instead of a complete database of fingerprints. When considering practical impact in realistic scenarios, multilateration may, thus, be a more suitable approach as it does not have the same privacy concerns.

If it is possible, given the device capabilities and constraints, to continuously sample the accelerometer and gyroscopic data together with the RSSI measurements, sensor fusion is a viable approach, as shown in the studies by [62,63]. Sensor fusion requires the same beacon database as multilateration uses, i.e., only beacon locations are needed, resulting in modest database sizes. When we used an unscented Kalman filter to combine PDR with the RSSI measurements, it was highly effective at compensating the sparse beacon advertisements and achieved higher accuracy than fingerprinting and multilateration. While using only the collected data, without augmentation, the sensor fusion method was still more accurate than both the augmented variant of multilateration and fingerprinting.

In other words, for privacy-aware users that do not want to share their location, all three methods can technically be run on-device to calculate user position. However, whereas fingerprinting is easy to implement, it requires substantial amounts of stored reference data covering most, if not all, possible locations. In contrast, multilateration and sensor fusion only require known beacon locations. Of these two, sensor fusion achieves much higher accuracy in real-life conditions where beacon samples are sparse, making it the preferred method if it is possible to sample sensor data. Furthermore, if the positions of each beacon could be embedded in the actual beacon advertisements, privacy would additionally increase. This could be possible in both the multilateration and sensor fusion methods and would completely remove the need for downloading a database, making the system entirely passive and privacy-preserving. The drawback would be that the person installing the system would have to know the exact location of each beacon at installation time instead of measuring it.

As we mentioned at the beginning, contact tracing for COVID-19 has become common, and many different applications can be downloaded for this purpose. Sowmiya et al. [64] wrote a survey of the security and privacy aspects of contact tracing applications. However, most approaches are based on all devices transmitting randomised IDs at all times, with one exception being the approach by [65], where RFID tags and receivers are used and the information is stored in a blockchain to preserve anonymity. A possible similar approach could be to, with user consent, upload the time and coordinates of when a COVID-19-infected person visited a specific location to the blockchain. If someone wants to know if his/she is likely to have been in contact with a contagious person, he/she could compare his/her local location history with information from the blockchain.

This paper evaluated indoor positioning methods that are often used with BLE beacons or WiFi access points. If we consider the number of new articles targeting UWB, which in our search amounted to 18% of all published articles between 2006 and 2021 (see Figure 1), we expect the privacy aspect to be of equal importance for that technology. In contrast to BLE broadcasting, many UWB protocols require two-way communication or complex infrastructures to enable positioning. This requirement poses an interesting challenge within UWB deployments, which we hope to target in future work. We also see Deep Neural Networks (DNNs) as possible candidates for replacing fingerprinting, especially if combined with knowledge distillation approaches such as by [66], to reduce the network size.

## 5. Conclusions

For indoor positioning, fingerprinting is the most-common approach and is often considered the gold standard. Due to storage and computational requirements, fingerprinting databases are often stored outside the user’s device, on a local server, or in the cloud. However, indirectly streaming the users’ location by submitting fingerprints is privacy-invasive. Given the complexity of making server-side location computations secure through, e.g., homomorphic encryption, we instead explored if passive sensor fusion can replace fingerprinting and multilateration methods for pedestrian indoor positioning. We validated this by setting up an experiment with commonly available hardware in a busy office building. Sensor fusion does not only require much smaller data storage, but can also give substantial accuracy improvements in busy environments. It can also be much faster to install, as it does not require complete fingerprinting databases. Our results showed that using a passive approach for indoor positioning is feasible and has the potential to respect the user’s privacy without compromises.

## Figures and Tables

**Table 1:** Rules processed top down to assign categories. The asterisk * matches any metadata.

Metadata Related to	Assigned Category
Fingerprinting and Multilateration	Fingerprinting and Multilateration
Ultra-Wideband	Ultra-Wideband
Multilateration	Multilateration
Fingerprinting	Fingerprinting
ML	Machine Learning
Sensor Fusion	Sensor Fusion
*	Other

**Table 2:** Number of published papers within indoor positioning 2006–2021.

Method	# of Papers	%
Fingerprinting	1177	32
Fingerprinting and Multilateration	116	3
Machine Learning	159	4
Multilateration	329	9
Other	727	20
Sensor Fusion	563	15
Ultra-Wideband	657	18
Total	3728	

**Table 3:** Attack types as described by [29].

Type of Attack	
Location Privacy Attack I	The attacker obtains client position directly from the query
Location Privacy Attack II	The attacker infers client position from the client RSSI data
Data Privacy Attack I	The attacker obtains the fingerprinting database from the server
Data Privacy Attack II	The attacker obtains a fingerprinting database that is similar to the one stored on the server

**Table 4:** Comparison between the three indoor positioning methods.

	Sensor Fusion	Multilateration	Fingerprinting
**Downloaded data**	Beacon positions	Beacon positions	Fingerprinting database
**Uploaded data**	None	None	None
**Sensor data collection**	Accelerometer 3-axis @ 50 Hz Gyroscope 3-axes @ 50 Hz Magnetometer 3-axes @ 20 Hz	None	None
**On-device calculations**	Medium	Medium	Very low
**Database size**	Small	Small	Large

**Table 5:** Location errors in m at CDF = 50% and CDF = 80%.

	Augmented			
	No		Yes	
**Method**	**CDF50**	**CDF80**	**CDF50**	**CDF80**
UKF + PDR	1.58	2.23	-	-
Multilateration	3.03	4.37	1.64	2.71
Fingerprinting	3.19	5.09	1.82	3.04

## Data Availability

The data presented in this study are available on request from the corresponding author.
